# Quantitatively Elucidating the Trade-Off between Zwitterionic Antifouling Surfaces and Bioconjugation Performance

**DOI:** 10.1021/acs.langmuir.4c03827

**Published:** 2024-11-21

**Authors:** Pai-Jung Yang, Yu-Ching Hsu, Jie-Ren Li, Shyh-Chyang Luo

**Affiliations:** † Department of Materials Science and Engineering, National Taiwan University, No. 1, Sec. 4, Roosevelt Road, Taipei 10617, Taiwan; ‡ Department of Chemistry, National Cheng Kung University, No.1, University Rd., Tainan 70101, Taiwan; § Institute of Polymer Science and Engineering, National Taiwan University, No. 1, Sec. 4, Roosevelt Road, Taipei 10617, Taiwan

## Abstract

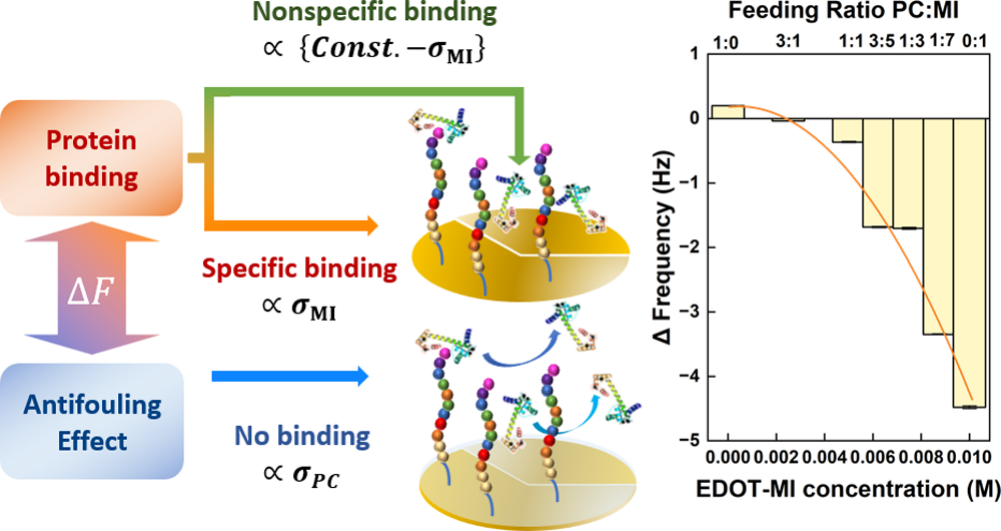

Zwitterionic materials, known for their high hydrophilicity, are widely used to minimize the nonspecific adsorption of biomolecules in complex biological solutions. However, these materials can also reduce the capture efficiency between targets and peptide probes. To demonstrate how antifouling surfaces affect capture efficiency, we utilize a poly(3,4-ethylenedioxythiophene) (PEDOT)-based surface incorporating varying ratios of phosphorylcholine (PEDOT-PC) and maleimide functional groups to achieve both antifouling properties and peptide–protein binding. As a model system, the peptide YWDKIKDFIGGSSSSC, attached via maleimide groups, is used to capture the target protein, calmodulin (CaM). By systematically monitoring protein binding on both antifouling and peptide-immobilized PEDOT surfaces using a quartz crystal microbalance with dissipation, the results reveal that PEDOT-PC reduces both the specific binding between peptides and target proteins as well as the rate of protein fouling on the electrode surface. From these findings, we propose an equation for quantitative analysis. Furthermore, electrochemical impedance spectroscopy and differential pulse voltammetry are performed to measure the changes in the impedance in CaM solutions. The data indicate that impedance increases with protein adsorption, confirming the practical utility of the designed electrode surface.

## Introduction

An electrochemical biosensor is a device that detects biological information by measuring electrical signals, such as the change in impedance or redox potential signal of a specific kind of substance.^1,2^ Data collected by the device allow us to analyze conditions within biological environments. Due to its portability, high sensitivity, and remote control capabilities, it is widely used in various fields, including clinical diagnostics, environmental monitoring, and the food industry.^1,3^ The sensor mechanism generally involves a particular kind of target binding on the electrode.^4^ The targets include DNA, RNA, protein, and so on.^5,6^ Therefore, the electrode usually needs a probe to capture specific agents and a substrate that provides a high conductivity to support the probe molecules.

Regarding probe candidates, traditional recognition elements include antibodies, enzymes, polymers, and cells. Recently, aptamers—whether DNA, RNA, or peptides—that bind to target agents and are selected from libraries of random sequence amino acids and nucleic acids have drawn significant scientific interest for their lower cost and higher specificity.^7,8^ Peptide aptamers have gained increasing attention in research due to their advantages of rigidity from secondary structures and chemical diversity offered by the wide variety of amino acids.^9^ Due to the advantage mentioned above, the peptide aptamer sequence YWDKIKDFIGGSSSSC was chosen as a probe based on its target protein, calmodulin (CaM), and the requirement of the cysteine terminate group to enable immobilization on substrates with maleimide groups.^10^

Materials with high conductivity and low impedance are needed to enhance the electrode sensitivity. However, traditional conductors like metals are often unsuitable for body tissues due to their high mechanical strength and poor biocompatibility.^11^ Conducting polymers have gained attention as promising alternatives because their mechanical strength is comparable to that of the extracellular matrix.^12^ Among them, poly(3,4-ethylenedioxythiophene) (PEDOT) is notable for its exceptionally low impedance, even outperforming platinum, thanks to its superior ionic conductivity.^13,14^ Additionally, PEDOT-based substrates with maleimide modification can connect with peptide aptamers with the cysteine group owing to thiol-maleimide click reaction.^10^ Therefore, a PEDOT-based substrate was selected for the electrochemical biosensor in our study.

However, the biosensor always faces a problem called nonspecific fouling. In the complex biological environment, many biomolecule adhesions on electrode surfaces induce immunological response and decrease the specificity of the electrode.^15,16^ Therefore, nonspecific adsorption of biomolecules, except targets, should be reduced. Around 1970, Davis concluded that the materials controlling the immunogenicity of proteins all have strong interactions with water.^17^ The reason is that the hydration layer formed on the surface with strong hydrophilicity prevents biomolecule adhesion. Zwitterionic materials, which are composed of positive and negative charged groups but keep their charge neutrality, construct a strong electrostatic attraction to water molecules.^18^ With the hydration layer adsorbed on the surface modified with zwitterionic materials, biomolecules have difficulty adhering to the surface. Since the early 2000s, Jiang has focused on advancing the study of zwitterionic materials. His research has uncovered vital strategies for achieving ultralow fouling in complex environments by fine-tuning the length, density, and thickness of zwitterionic groups.^19,20^ Furthermore, molecular simulations have provided more profound insights into the mechanisms underlying the interactions between zwitterionic groups and water molecules.^21^ Building on the strong foundations established by earlier pioneers in the field, there has been a surge of recent studies focused on zwitterionic materials.^22−25^ These studies have explored the diverse applications and morphologies of zwitterionic materials across various fields. For instance, zwitterionic micelles have been investigated for water–oil separation technologies^26^ and drug delivery system^25,27^ owing to dispersion stability in an aqueous environment. Due to excellent nonfouling properties and biocompatibility, zwitterionic ultrathin film^28^ and hydrogel^29^ have also been employed in biomedical applications. The antifouling and antimicrobial coatings help to prevent the accumulation of unwanted biological matter and reduce bacterial growth on surfaces.^30,31^ In addition, novel zwitterionic molecule designs have popped out in the past few years, such as zwitterionic peptide with EK sequence^32^ and cell membrane mimicking the phosphorylcholine group. Both of these designs demonstrate a prominent antifouling property to biomolecules and are commonly used in an electrochemical biosensor.^33,34^ Considering the precise control of proportion between antifouling material and peptide probes, we eventually chose the phosphorylcholine group as antifouling material to prevent the kinetic competition between the EK sequence and peptide probe.

Nevertheless, the specific binding between probes and target agents can be influenced by antifouling materials, potentially affecting the performance of the electrochemical biosensors. Despite this, there are a few systematic studies discussing this issue. In our studies, we aim to analyze the effect of antifouling materials on specific capturing using a quartz crystal microbalance with dissipation monitoring (QCM-D), an instrument measuring the frequency change associated with mass variation. However, although mass variation indicates the amount of protein adsorption, it does not reveal the underlying adsorption mechanism. To overcome this technical limitation, we designed an electrode with varying ratios of probes to antifouling materials to quantitatively separate the effect of antifouling on specific adsorption. Two kinds of EDOT derivatives, EDOT-MI and EDOT-PC, were copolymerized on a gold surface to attach peptide aptamers and provide nonfouling properties, respectively. Keeping the total feeding concentration of EDOT-MI and EDOT-PC constant, the molar concentration of EDOT-PC is expressed in terms of EDOT-MI. In this approach, the frequency measurements obtained via QCM-D can be represented as a quadratic function of the EDOT-MI feeding concentration. By developing a mathematical model that describes the relationship between antifouling effects and protein binding through two distinct mechanisms, we illustrate the impact of antifouling materials on the efficiency and sensitivity of electrochemical biosensors.

## Materials and Methods

### Materials

Phosphate-buffered saline (PBS) tablets and calmodulin (CaM) protein were purchased from Sigma-Aldrich. Potassium hexacyanoferrate trihydrate and anhydrous lithium perchlorate (LiClO_4_) were purchased from Alfa Aesar. Potassium ferricyanide and dioctyl sulfosuccinate (DSS) were purchased from Acros Organics. Tetrabutylammonium perchlorate was purchased from TCI CO., Ltd. Acetonitrile was purchased from Fisher Scientific Korea Ltd. Hydrogen peroxide was purchased from Fluka-Honeywell. Sulfuric acid was purchased from Showa Chemical Industry Co., Ltd. EDOT-OH was purchased from Angene Chemical. The chemicals mentioned above were used directly without further purification. EDOT-MI and EDOT-PC were synthesized according to the previous study.^11^ Peptide YWDKIKDFIGGSSSSC (purity = 90.25%) was purchased from Yao-Hong Biotechnology Inc.

### Electropolymerization of Poly(EDOT-MI-*co*-EDOT-PC)

10 mM of EDOT-PC and EDOT-MI solution was mixed together at different ratios in AOT (a solution with anhydrous acetonitrile as solvent, 50 mM DSS as surfactant to assist hydrophilic EDOT-PC to dissolve in an organic environment, and 100 mM LiClO_4_ as electrolyte). Poly(EDOT-MI-*co*-EDOT-PC) was electropolymerized on QCM chips (QSX 301 Au chip, diameter 14 mm, Biolin Scientific Co. Ltd.), which is the working electrode via standard three-electrode system with an Autolab potentialstat (PGSTAT128N) from solutions with different feeding ratio of EDOT derivatives in AOT at 20 °C. An Ag/Ag^+^ reference electrode was used in the organic solvent, and a platinum wire was taken as a counter electrode. The QCM chips were cleaned with piranha solution containing H_2_SO_4_ and H_2_O_2_ with a volume ratio = 3:1, washed the solution with DI water, and dried with air previously. Positive constant potential 1.1 V vs Ag/Ag^+^ was applied for 5 s to oxidize monomers and proceed with copolymerization on the working electrode. Then, the copolymer was reduced at a negative potential of −0.5 V for 3 s. The current–time and charge-time data are shown in Figure S1.

### QCM Measurement

The fouling of biomolecules was measured through a QCM-D E1 (Biolin Scientific, Sweden) system under 25 °C quantitatively to compare the interaction among different copolymer films, peptide aptamers, and CaM. Poly(EDOT-MI-*co*-EDOT-PC) was electropolymerized on QSX 301 Au chips with different feeding ratios and placed in the chambers of the QCM-D system. Buffer and biomolecule solutions were pumped through chambers at a 30 μL/min flow rate via an Ismatec ISM597D pump. With biomolecules binding on the QCM chip surface in the chamber, the resonance rate decreases and leads to frequency change (Δ*f*) related to the mass increase on the chip. Seven overtones were recorded by QCM-D E1 (*n* = 1, 3, 5, 7, 9, 11, and 13), and only the third overtone (*n* = 3) was shown in this study. To minimize the frequency change led by the buffer of biomolecule solutions, a stable baseline with a frequency change under 2 Hz within 30 min under pure buffer was set before the measurement.

### AFM Characterization

The local roughness and thickness measurements were conducted using MultiMode 8 atomic force microscopes (AFM) with a NanoScope-V controller (Bruker, Santa Barbara, CA, USA) under tapping mode with a typical force less than 1 nN. A rectangular silicon cantilever with a sharpened probe (tip radius of less than 5 nm), an average force constant of 48 N/m, and an average resonance frequency of 190 kHz (PPP-NCLR, NANOSENSORS, Neuchatel, Switzerland) was used for tapping mode AFM measurements of samples. To measure the film thickness, an area of the surface was shaved with a square area of 1 × 1 μm^2^ on the sample by AFM-based nanoshaving, which is accomplished by applying mechanical force to the AFM probe during scans. A high force (40–60 nN) was applied to the AFM tip, while a selected region was scanned to sweep away the film molecules under the tip. Information about the film thickness can be obtained by using the cursor profiles of nanoshaved areas as a baseline for cursor measurements. Cursor profiles from topography and morphology analysis were acquired from AFM data with NanoScope Analysis software (version 1.9, Bruker, Santa Barbara, CA, USA) and Gwyddion (version 2.52), an open source software supported by the Department of Nanometrology, Czech Metrology Institute.

### Electrochemical Measurements

Electrochemistry impedance spectroscopy (EIS) and differential pulse voltammetry (DPV) measurements were conducted by using a potentiostat (PGSTAT128N, Autolab). A sinusoidal potential with an amplitude of 10 mV at frequencies spacing from 10 kHz to 1 Hz was applied to measure the impedance of copolymer coated on Au surfaces in EIS measurement. DPV measurement swept linearly from −0.2 to 0.6 V and monitored the current before the potential change. With surface modification, the current peak might decrease or increase according to impedance changes. The copolymer films are coated on the Au surface (Area = 1 cm^2^). The gold electrode as a working electrode, an Ag/AgCl reference electrode, and a Pt wire as a counter electrode were installed as the three-electrode system in Fe^2+^/Fe^3+^ solution at 20 °C. The solution contained 10 mM K_3_Fe(CN)_6_ and K_4_Fe(CN)_6_ as redox pair, 100 mM KCl as electrolyte, and PBS as solvent.

## Results and Discussion

To investigate whether antifouling materials affect the bioconjugation and the detection efficiency of electrochemical biosensors, we need to design a model and method to differentiate the antifouling effect on the nonspecific and specific binding of target biomolecules. With a quartz crystal microbalance of dissipation (QCM-D), the number and mass of proteins adsorbed on surfaces can be quantized, but it was hard to identify the binding pathway. Fortunately, we can solve this problem via experimental design.

**Figure 1:**
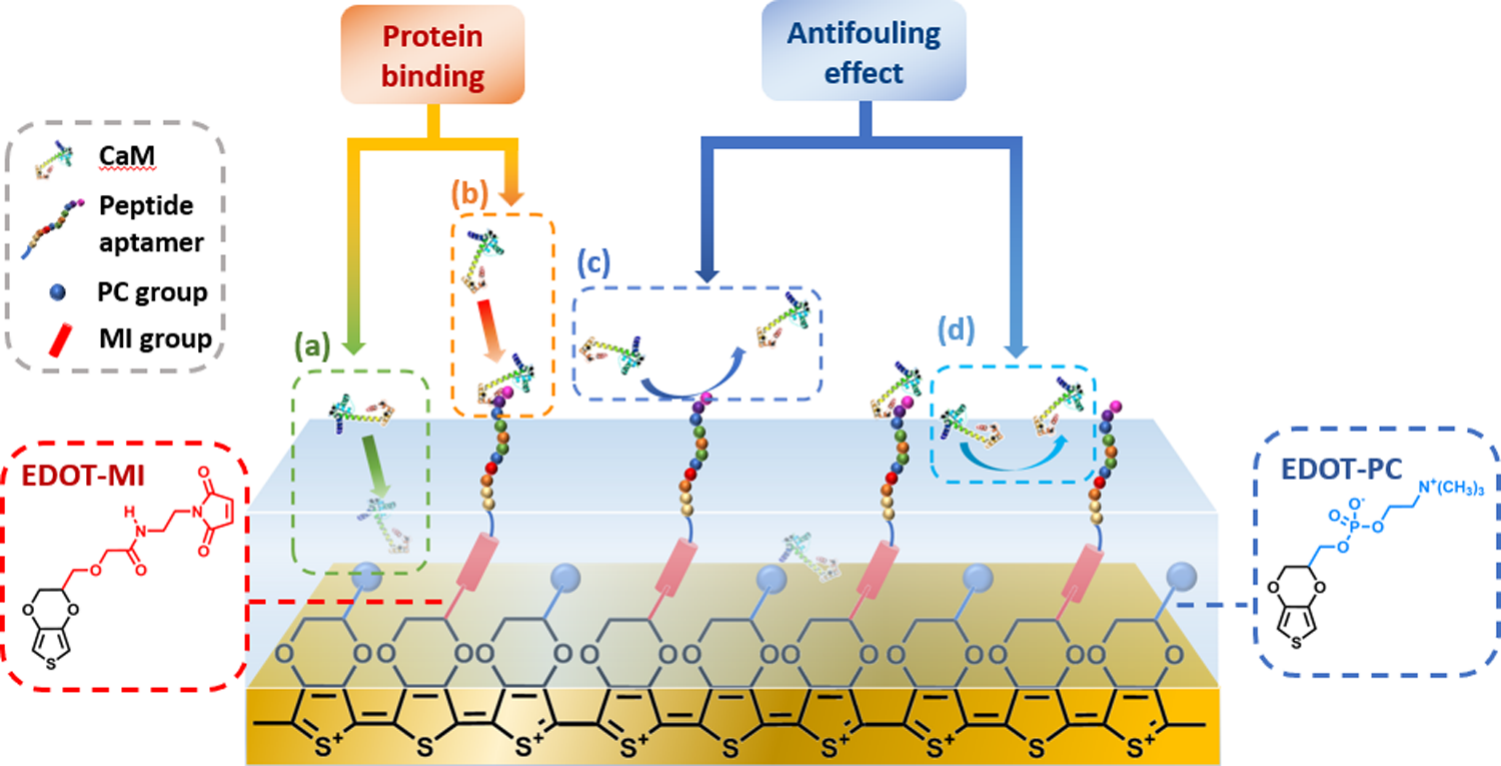
Electrode design and the different protein interaction modes with the electrode include (a) nonspecific binding, (b) specific binding, (c) the antifouling effect on reducing specific binding, and (d) the antifouling effect on preventing nonspecific binding.

The electrode design requires a low impedance to facilitate sensibility on an electrochemical biosensor, an ability to catch target biomolecules, and a high hydrophilicity. Therefore, PEDOT-based surfaces were constructed with varying proportions of zwitterionic phosphorylcholine and maleimide functional groups (Figure 1). As we mentioned in the introduction section, the electrochemical impedance of PEDOT-based surfaces is even lower than traditional conductive metals, such as platinum and gold. For the purpose of detecting specific target biomolecules, peptides are versatile probes to distinguish the target from complex environments such as serum. Hence, in this model, with a maleimide group modification on PEDOT molecules, peptides of CaM protein, whose sequence is YWDKIKDFIGGSSSSC with cysteine amino acids as end groups, can connect to maleimide groups through a thiol-maleimide click reaction. Zwitterionic phosphorylcholine groups with polarity, which can bond with water strongly, were adopted to enhance the antifouling property. With the water layer adsorbed on the chips, it is difficult for biomolecules to be adsorbed on the surface.

With this electrode surface design, the proteins conjugated with peptides are proportional to the EDOT-MI surface concentration after saturation, according to the monolayer model. Conversely, the nonspecific binding proteins are proportional to the rest of the surface. In addition, we propose that the number of proteins adhered on the surface under the antifouling effect is also proportional to the antifouling EDOT-PC surface concentration. In order to represent the EDOT-PC surface concentration as a function of the EDOT-MI surface concentration, the sum of the EDOT-PC and EDOT-MI surface concentrations is manipulated as a constant. Based on the assumption above, the frequency change measured via the QCM-D of CaM on the designed surface is the sum of two terms. One is the specific binding protein. If the antifouling effect impacts this term, it may be proportional to the product of (const.– [EDOT-MI]) × [EDOT-MI]. The other term is nonspecific binding under the influence of the antifouling effect, which may be related to the product of (const.– [EDOT-MI]) × (const. – [EDOT-MI]). Hence, the fitting of the frequency change to [EDOT-MI] might be a quadratic equation with a positive or negative coefficient of the second-order term. If the coefficient is negative, it indicates that the antifouling property affects specific binding. The detailed calculation is summarized in Table 1.

**Table 1:** Relation and Detailed Calculation of the Antifouling Effect on Two Protein Dissipation Mechanisms

	protein binding	antifouling	Δ*f* = protein binding × antifouling	*x*^2^ coefficient
nonspecific	const. – *x*	const. – *x*	(const. – *x*)^2^	+
specific	*x*	const. – *x*	*x* × (const. – *x*)	–

**Figure 2:**
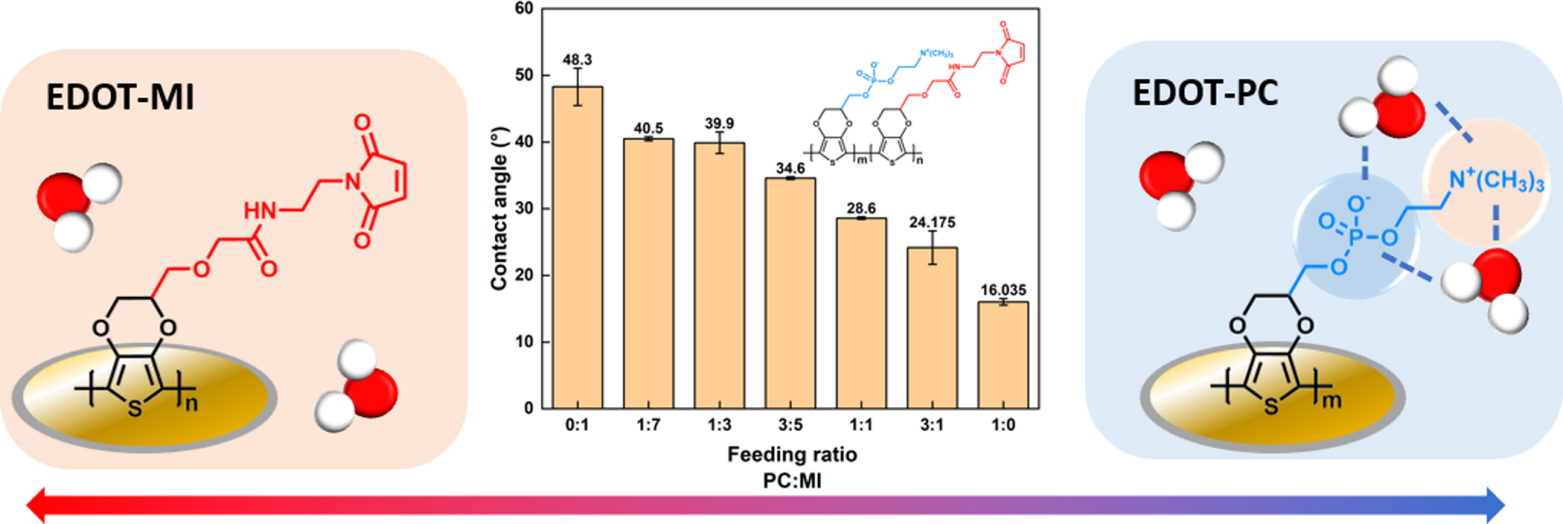
Contact angles of poly(EDOT-MI-*co-*EDOT-PC) film with different feeding ratios.

To prepare the electrode, we blended 10 mM EDOT-MI and 10 mM EDOT-PC solution. The volume fraction was manipulated so that the molar concentration of EDOT-PC can be represented as 10 mM – [EDOT-MI]. To confirm that the EDOT derivative monomers are electropolymerized on the surface, contact angles of water on the chips were measured. Figure 2 shows the contact angle of water on chips with poly(EDOT-MI-*co*-EDOT-PC) copolymerized under the different feeding ratios of EDOT-MI and EDOT-PC. With pure PEDOT-MI on the surface, the average contact angle was about 48.3°, and the average contact angle on the pure poly(EDOT-PC) surface was about 16.0°. The contact angle gradually reduced with an increasing concentration of phosphorylcholine groups and decreasing concentration of maleimide groups. According to the linear fitting shown in Figure S2, the contact angles declined by 3.7° with an increase of 1.25 mM EDOT-PC and a reduction of 1.25 mM EDOT-MI. The result corresponds to our anticipation that the phosphorylcholine group prompts hydrophilicity due to the electrostatic attraction between water and the zwitterionic functional group. In addition, the measurement ensures that there is no significant difference between the proportion of two functional groups on the electrode surface and the feeding ratio. In addition, based on the roughness measured by atomic force microscopy (AFM) demonstrated in Table S1a, the roughness of pure poly(EDOT-PC) film and copolymer films seemed generally smooth and would not affect contact angles significantly. The film thickness is shown in Figure S3, ranging from 1 to 2.5 nm, an extremely thin layer.

**Figure 3:**
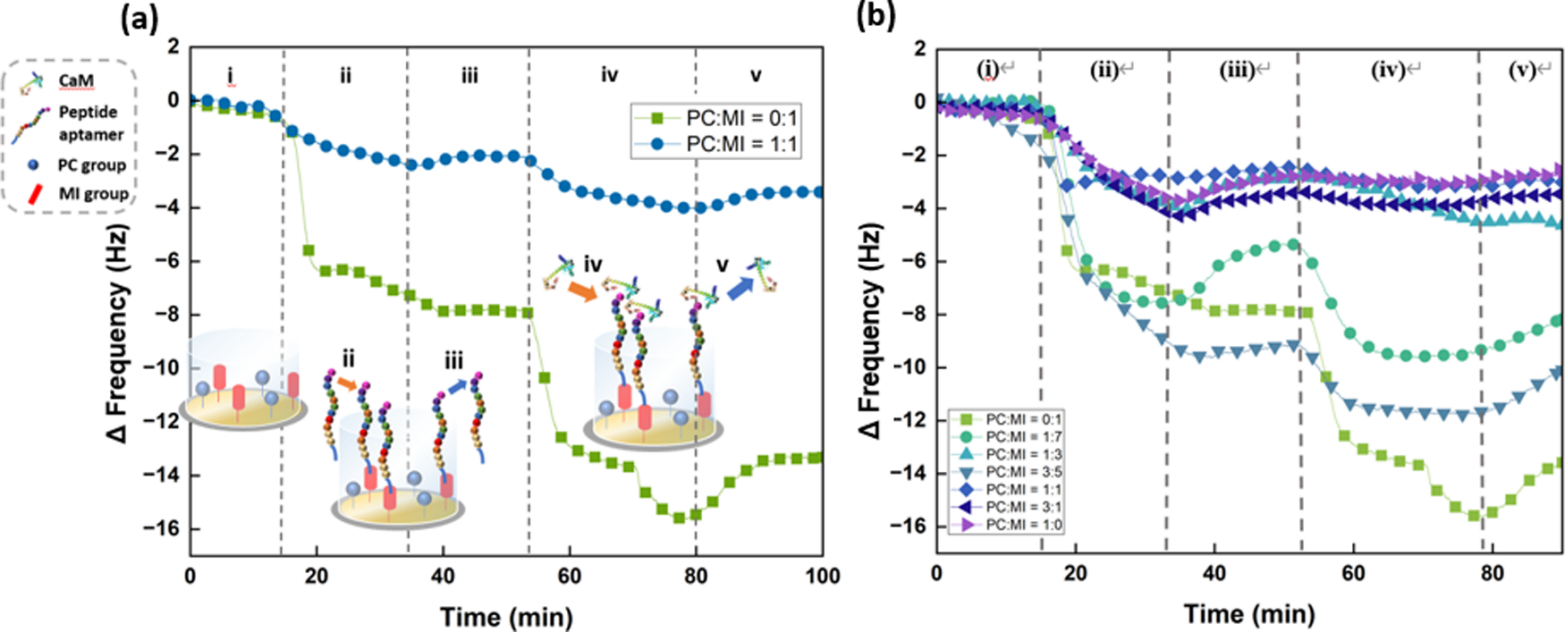
(a) Procedure and real-time monitoring of the reactions of peptides and proteins with poly(EDOT-MI-*co*-EDOT-PC) film. (i) Equilibrium reached under the buffer flow. (ii) Peptide probe immobilization. (iii) Rinsing chips with buffer containing Ca^2+^. (iv) CaM protein bioconjugation with the peptide probe. (v) Rinsing chips with buffer containing Ca^2+^ again. (b) Real-time frequency change on the Au surfaces with copolymer films composed of different feeding ratios of EDOT-MI and EDOT-PC during the QCM-D measurements.

The peptide conjugation through thiol-maleimide click reaction, CaM binding on peptide aptamer, and nonspecific adsorption on the surfaces are confirmed in situ via QCM-D. In Figure 3a, the stages of the experiment are demonstrated. The frequency measured through QCM-D reached equilibrium under the flow of PBS (pH = 6.0). Then, the peptide aptamers were dissolved in a buffer at a 0.1 mg/mL concentration and reacted for 20 min. The chips were rinsed with PBS with saturated calcium ions, which transformed the conformation of calmodulin to Ca^2+^-CaM. With this new conformation, the peptides (YWDKIKDFIGGSSSSC) can catch the target protein Ca^2+^-CaM. Therefore, at stage (iv), after calcium ions were added to the buffer and reached a new balance with the surface for 20 min, 20 mg/L CaM with saturated Ca^2+^ ions substituted the blank buffer and interacted with the chip surfaces for 25 min. Lastly, the chambers were rinsed with a blank buffer with saturated Ca^2+^ ions again to wash out the proteins that were not tightly bound on the surface. More real-time monitored data with different ratios are shown in Figure 3b. According to the AFM data shown in Table S1b, the roughness of the surface equals 2.3 nm after the peptide solution flew through the chamber, which is higher than that of the original copolymer film. In addition, the surface roughness with peptide probes increased to 4.9 nm after protein binding. These indicate that the surface becomes rougher after biomolecule adhesion and immobilization.

Comparing the frequency change of the peptides binding on poly(EDOT-MI*-co-*EDOT-PC) film with the different feeding ratio (Figure S4), the frequency drop of the samples is closed (∼2–3 Hz) except for the one of pure poly(EDOT-MI) (∼6.6 Hz). Strangely, the number of peptides on the surface does not change significantly with the different maleimide group concentrations except the one with pure poly(EDOT-MI). Even in the absence of the maleimide group, peptide adsorption on the surface is still observed. Moreover, regarding protein adsorption, we observe that it increases with the density of maleimide groups, even though the levels of peptide dissipation remain similar. There is a possible explanation. In the peptide sequence, two charged amino acids exist. They are aspartic acid (D), which carries a negative charge, and lysine (K), an amino acid with a positive charge. The phosphorylcholine group may attract these charged amino acids due to electrostatic attraction force. However, due to its wrong orientation, the peptide linked to phosphorylcholine cannot serve as the active site for CaM conjugation. Hence, even with a similar number of peptide aptamers on the surfaces, there are more peptides with the correct orientations on the sample with more maleimide groups than those with more phosphorylcholine groups. In this case, the number of peptide probes with the proper orientation can still be estimated, which is proportional to the concentration of the maleimide functional group.

**Figure 4:**
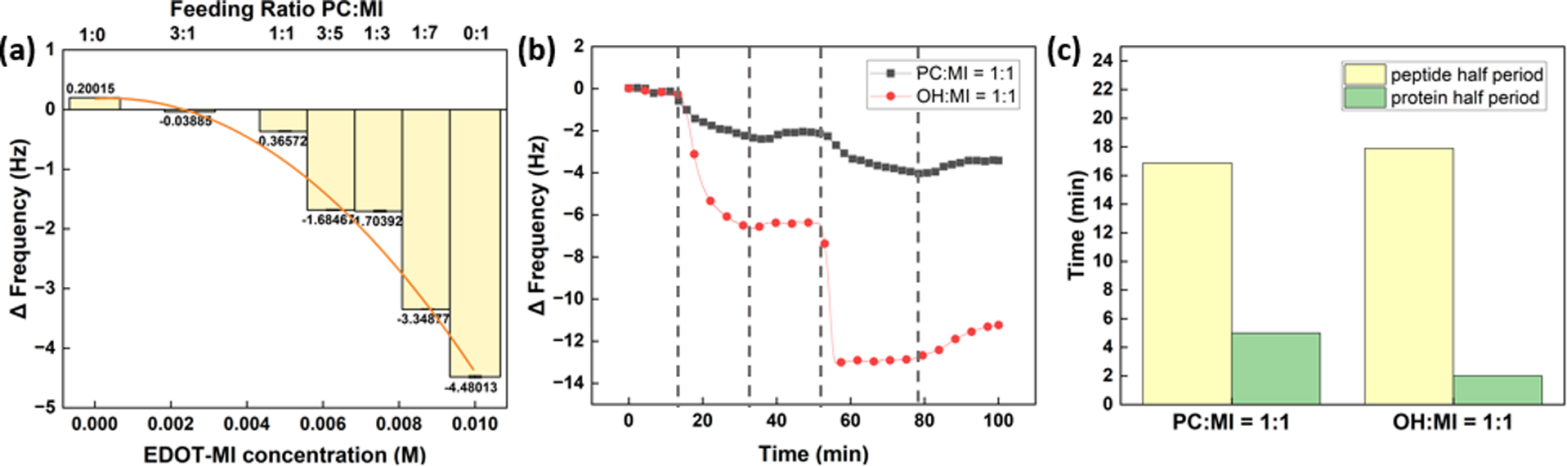
(a) Frequency change of protein adsorption on the surfaces with different maleimide and phosphorylcholine feeding concentrations and its fitting. (b) Real-time monitoring of peptides and protein adhesion via QCM-D. (c) Half-time of the maximum frequency drop led by peptide and protein adsorption on the surface of poly(EDOT-MI-*co*-EDOT-PC) and poly(EDOT-MI-*co*-EDOT-OH) was reached.

The frequency change between 55 and 90 min of different groups was recorded in Figure 4a. At 55 min, the peptides were washed from the chips, and the protein solution started to flow into the chamber. After 25 min, the protein solution was replaced by the blank buffer with calcium ions to rinse the chamber for 10 min, 90 min from the beginning of the experiment. Therefore, the frequency drop during this period can be viewed as the data representing the CaM adsorption.

The frequency change led by protein adsorption on the pure poly(EDOT-PC) film is about 0.2 Hz. The reason for the frequency increase, which meant that the total mass on the chips became lighter, was that nearly no protein was bound on the surface, and some peptides were flushed from the surfaces during this period. It demonstrates that the phosphorylcholine group is an outstanding antifouling material, almost preventing protein adhesion through nonspecific binding. The maximum frequency drop (∼4.5 Hz) happens in the group with the pure poly(EDOT-MI) film. The protein adsorption on the copolymer film gradually inclined with increasing maleimide and decreasing phosphorylcholine surface concentration. With more maleimide groups and less zwitterionic antifouling phosphorylcholine, the number of specific and nonspecific binding proteins grew and reached the maximum on the poly(EDOT-MI) film simultaneously. The data were fitted with the second-order polynomial model by setting the maleimide feeding molar concentration as an independent variable and frequency change as a dependent variable. The equation and its coefficients are shown in Table 2, with *R*^2^ equal to 98.192. The value of *R*^2^ indicates that the equation is a reliable regression model to the data and corresponds to the assumption of the relation between the frequency change and EDOT-MI feeding concentration. In addition, the intercept of the quadratic equation is nearly zero. Due to the strong antifouling property, the value expresses that there is almost no protein adhesion on the surface with maximum phosphorylcholine and no maleimide groups. Although the fact that the coefficient of the first order term equals 37.93465 ± 59.6765 Hz M^–1^ points out that the maximum quadratic equation does not happen when the film consists of pure poly(EDOT-PC) film, zero is included in the error value range, which indicates the maximum point of the frequency change may pass the point with the most phosphorylcholine surface concentration. With the cues above, the fitting model reasonably explains the experiment results.

**Table 2:** Fitting Equation of the Relation between the Frequency Changes Measured through QCM-D and the EDOT-MI Feeding Concentration and Related Information

equation (Hz)	*Y* = intercept + B1 × *x* + B2 × *x*^2^
intercept (Hz)	0.17309 ± 0.11218
B1 (HzM^–1^)	37.93465 ± 59.6765
B2 (HzM^–2^)	–48819.16208 ± 6750.20142
R-square (COD)	0.98182
adj. R-square	0.97273

Focusing on the main objective of this project, we note that the coefficient for the second term is negative (∼ −48819.16 Hz M^–2^), even when the error value is considered (∼6750.20 Hz M^–2^). According to the assumptions we have discussed, this negative coefficient suggests that hydrophilic phosphorylcholine groups affect the bioconjugation between peptides and CaM. This finding indicates that the antifouling effect may influence specific binding, though the impact can vary depending on the situation. Factors such as the antifouling capability and hydrophilicity of the zwitterionic material, the thickness of the water layer adsorbed on the surface, and the height of the peptides could all influence whether antifouling materials affect specific binding. Additionally, we face the challenge of balancing enhanced antifouling properties to prevent nonspecific adsorption against the potential loss of specific binding efficiency or sensitivity. These issues warrant further investigation in future studies.

In order to investigate if the kinetics of bioconjugation can also be impacted by antifouling material, we prepared a control group substituting phosphorylcholine to hydroxyl groups, and the feeding ratio of both groups equals 1:1. The frequency change monitored through QCM-D is illustrated in Figure 4b. The graph demonstrates that the frequency drops of both peptides and protein adhesion on the poly(EDOT-MI-*co*-EDOT-OH) film were both significantly higher than those on the poly(EDOT-MI-*co*-EDOT-PC) film. Without zwitterionic material and the water layer on the surface, the frequency drop increases due to the contribution of both nonspecific and specific binding peptides and proteins. To compare the kinetics of bioconjugation between the two groups, the half-time that the frequency drop reached the maximum point is recorded in Figure 4c. The result shows that the half-time of peptide adhesion of both groups was similar, which was nearly 17 min. This result corresponds to the explanation that the zwitterionic groups may attract the peptides with charged groups. However, we should focus on the number of peptides that adhere to the surface. In that case, the one on the surface without antifouling material is still three times higher than the one with the phosphorylcholine group. It demonstrates that antifouling prevents some peptides from adsorbing on the surface. Comparing the half-time of protein adsorption of both groups, we can find that the film consisting of the phosphorylcholine group was about 5 min, much longer than the one of the control group (∼2 min). This result means that zwitterionic materials not only decrease the number of protein binding but also the adsorption rate. The probable explanation is that target biomolecules are hardly recognized on the antifouling surface since the water layer prohibits biomolecules from staying on the surface for an extended period.

In conclusion, antifouling materials impact both specific and nonspecific binding on the number and dynamics of biomolecules adsorbed on the surface. Therefore, when nonfouling materials are applied to the electrochemical biosensors to enhance the specificity, we should notice their impacts on detecting efficiency.

**Figure 5:**
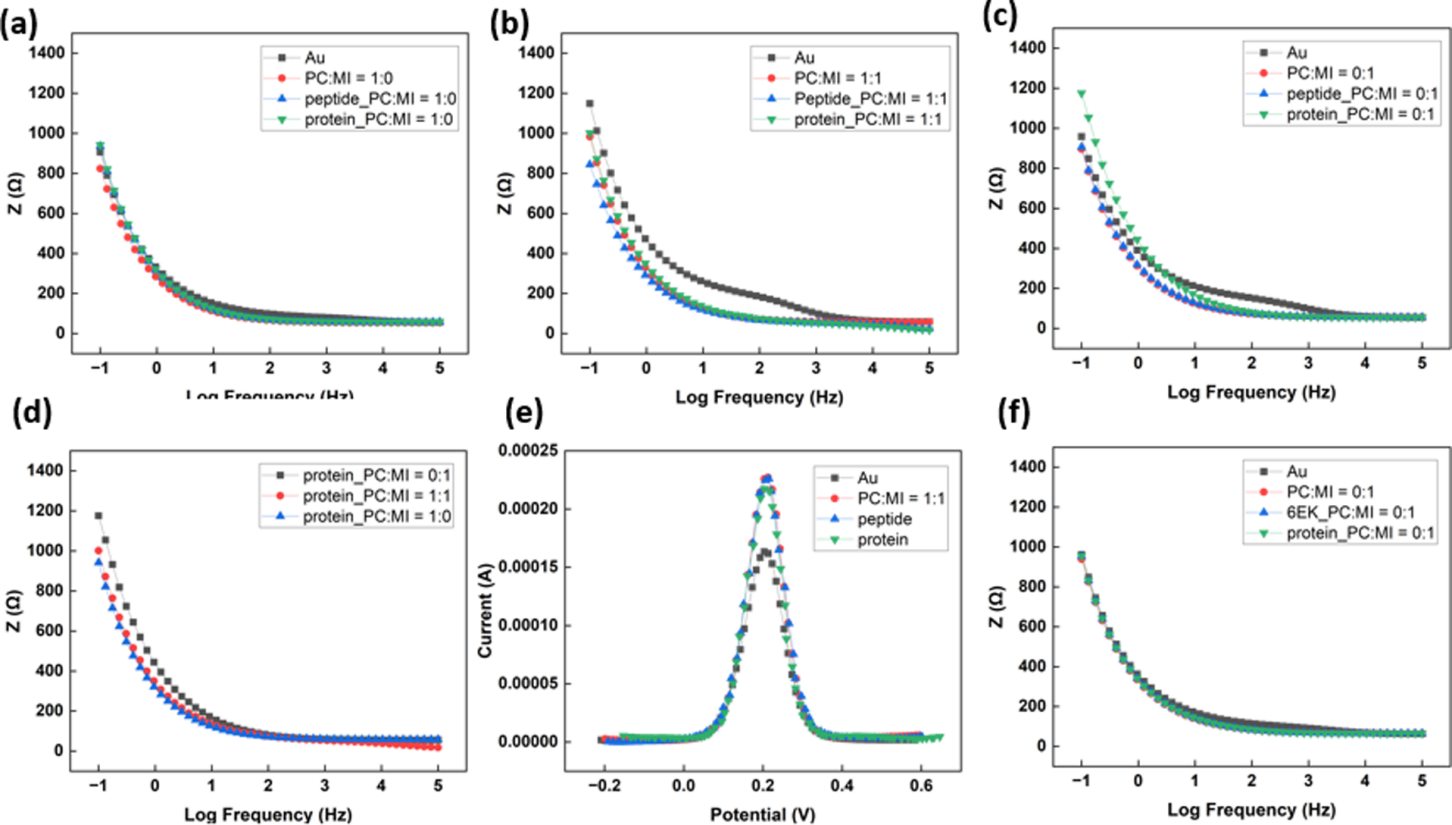
Electrochemical measurement. EIS measurement of different feeding ratios of EDOT-MI and EDOT-PC, which are equal to (a) 0:1, (b) 1:1, and (c) 1:0, respectively, and (d) their comparison after protein binding. (e) DPV measurement of different stages on the poly(EDOT-MI-*co-*EDOT-PC) film. (f) EIS measurement of the poly(EDOT-MI) film and the data after CPPPP(EK)_6_ reacted with the surface.

EIS and DPV measurements were applied to investigate the signal change when peptides and proteins react with the surface to ensure that the surface design works with electrochemical biosensors. The poly(EDOT-MI-*co*-EDOT-PC) film was electropolymerized on the gold electrodes. Then, the 0.1 mg/mL peptide solution was dropped on the electrode surface and reacted at 4 °C for 20 h. At last, the electrode with peptides reacted with 20 mg/L CaM in the PBS buffer with saturated Ca^2+^. The EIS and DPV measurements were conducted before every step, and the results are shown in Figure 5.

For the EIS measurement, the impedance of different feeding ratios is shown in Figure 5a–c. With EDOT derivatives electropolymerized on the gold surface, the impedance decreased significantly for all three groups. The extremely low impedance is contributed by the conductive conjugation π structure and the space between polymer chains that enable ions to exchange charges. What was worth paying attention to was the impedance, which remained almost the same or even lowered after reacting with peptides. The DPV signal also shows the corresponding trend, in which the peak currents do not decrease significantly (Figure 5e). Generally, with biomolecules adhered to the surface, the impedance increases due to the obstruction of ions from exchanging charge with electrodes. A possible explanation is that ion mobility is affected by the charge groups D and K in the peptide series. To investigate this situation, our peptides were replaced with CPPPP(EK)_6_, and the result is shown in Figure 5f. The impedance also showed no significant difference. The result indirectly supports the explanation that the zwitterionic functional group may facilitate ion conductivity. This situation is worth further study because if the peptides can lower or keep the impedance value with some design, they enhance the sensitivity of electrochemical biosensors.

Regarding the CaM detection, we found almost no change after the electrode with the poly(EDOT-PC) film reacted with CaM. In contrast, the impedance was raised on the other two films, which consist of maleimide functional groups that react with peptides and catch CaM. The impedance comparison between different film compositions after protein adsorption is demonstrated in Figure 5d. Integrated with the number of protein adsorption detected through QCM-D, the impedance increased with the number of proteins adsorbed on the surface. In addition, the DPV signal decreased after the electrode reacted with the CaM solution. This indicates that our electrode can successfully detect CaM through EIS and DPV methods. More information on EIS and DPV measurements on poly(EDOT-MI-*co*-EDOT-MI) are provided in Figure S5.

## Conclusions

In this study, we design an electrochemical biosensor surface with a PEDOT-based substrate modified by maleimide groups, which react with peptide aptamer probes, YWDKIKDFIGGSSSSC, and phosphorylcholine groups, which act as zwitterionic materials, to observe the antifouling effect on specific binding. Through the QCM-D, we measured the frequency drop of CaM adsorbed on the electrode surface. The fitting shows that the frequency change of CaM is a function of maleimide concentration equal to −48819.16208 × *x*^2^ + 37.93465 × *x* + 0.17309. The negative leading coefficient indicates that the phosphorylcholine groups impact the specific binding between peptides and CaM. In addition, we found that zwitterionic groups influence the dynamics of CaM adsorption, which might decrease the efficiency of the electrochemical biosensor. The EIS and DPV measurements ensure that the electrodes with zwitterionic groups are workable for sensing CaM because the impedance increases with the number of CaM fouling on the electrode. These results highlight the importance of carefully controlling and fine-tuning the antifouling composition while balancing its impact on the specific binding between the probes and targets. This approach could offer a promising pathway for improving the design of electrochemical biosensors in the future.

## Supporting Information Available

Current–time monitoring of electropolymerization process and water contact angle measurement, AFM images showing topography and adhesion mapping, real-time frequency and dissipation readouts of CaM binding by QCM-D, and the EIS and DPV measurement (PDF)

## Abbreviations

PEDOT: poly(3,4-ethylenedioxythiophene)
QCM-D: quartz crystal microbalance with dissipation
CaM: calmodulin protein
MI: maleimide functional group
PC: phosphorylcholine functional group
EIS: electrochemistry impedance microscopy
DPV: differential pulse voltammetry
