# Supplementary material for: Quantitatively Elucidating the Trade-Off between Zwitterionic Antifouling Surfaces and Bioconjugation Performance
Source: Langmuir. 2024 Nov 21;40(49):26259–66. doi: 10.1021/acs.langmuir.4c03827 (PMC11636210; doi:10.1021/acs.langmuir.4c03827)
Supplement: Supplementary file 1 — la4c03827_si_001.pdf [file la4c03827_si_001.pdf]

Supporting Information

Quantitatively elucidating the trade-off between zwitterionic antifouling surfaces and bioconjugation performance

Pai-Jung Yang,[a] Yu-Ching Hsu,[b] Jie-Ren Li,[b] and Shyh-Chyang Luo[a,c]**

[a]Department of Materials Science and Engineering, National Taiwan University, No. 1, Sec. 4, Roosevelt Road., Taipei 10617, Taiwan

[b]Department of Chemistry, National Cheng Kung University Department of Chemistry, No.1, University Rd., Tainan 70101, Taiwan

[c]Institute of Polymer Science and Engineering, National Taiwan University, No. 1, Sec. 4, Roosevelt Road., Taipei 10617, Taiwan

*Email: jierenli@ncku.edu.tw, shyhchyang@ntu.edu.tw

Table of Contents

Experimental Section
Figure S1: Electropolymerization of poly(EDOT-PC-*co*-EDOT-MI) on Au surface.
Figure S2: Linear fitting of contact angle.
Table S1: Roughness of surfaces measured by AFM.
Figure S3: The thickness measurement obtained from cursor profile and the AFM topography image.
Figure S4: The bar chart of peptides frequency drops and its possible mechanism.
Figure S5: The EIS and DPV measurement of poly(EDOT-PC-*co*-EDOT-MI) surface reacted with peptides and CaM.

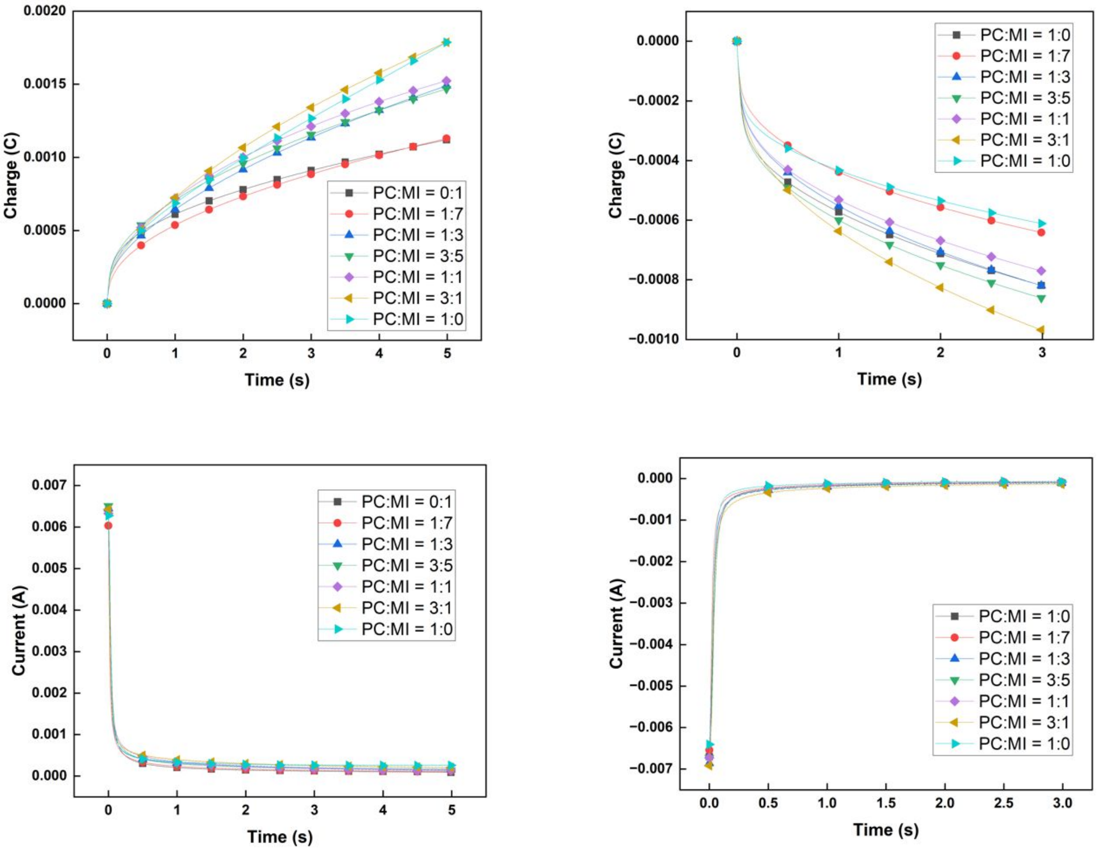

(a) **(b)**

Figure S1. The charge-time graph of electropolymerization (a) at 1.1 V and (b) at -0.5 V**.** The current-time graph of electropolymerization (c) at 1.1 V and (d) at -0.5 V.

(c) **(d)**

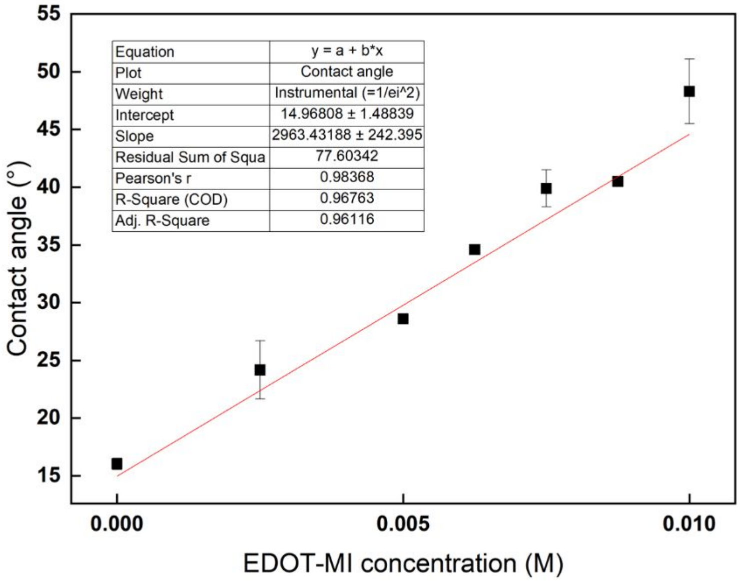

Figure S2. The linear fitting of the relation between contact angles and EDOT-MI molar concentration.

Table S1. AFM roughness data of QCM chips with (a) Poly(EDOT-PC) and Poly(EDOT-MI-*co*-EDOT-PC) film without peptides and proteins adsorption and (b) Poly(EDOT-MI-*co*-EDOT-PC) films with feeding ratio of EDOT-MI and EDOT-PC equals to 3:1 in stage (i), (iii) and (v) of QCM measurement.

(a)

**Film**	**Roughness (nm)**	**Standard deviation**
Poly(EDOT-PC)	3.02	1.41
Poly(EDOT-MI-*co*-EDOT-PC)	1.07	0.07

(b)

**Film**	**Roughness (nm)**	**Standard deviation**
Poly(EDOT-MI-*co*-EDOT-PC)	1.07	0.07
After 40 minutes (peptides)	2.30	0.33
After 100 minutes (proteins)	4.94	2.00

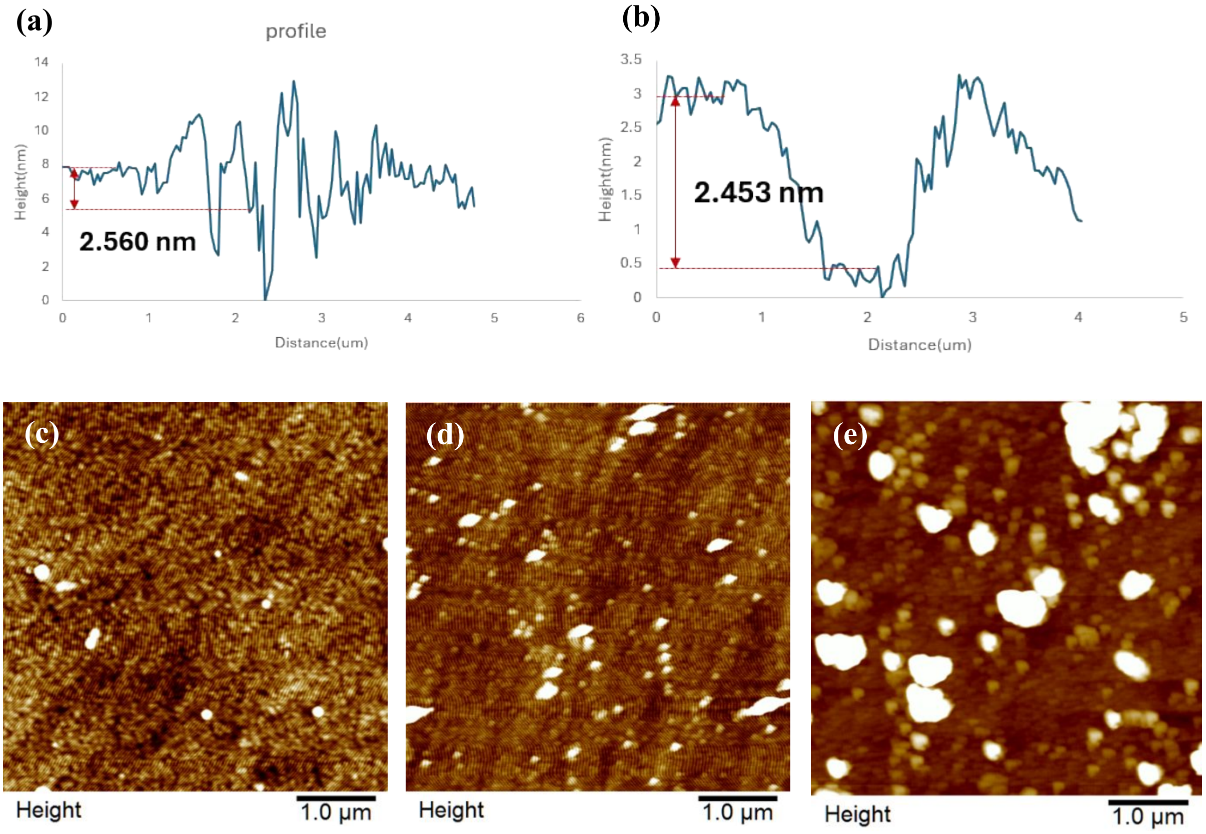

Figure S3. The thickness measurement was obtained from the cursor profile of (a) poly(EDOT-PC) and (b) poly(EDOT-MI-*co*-EDOT-PC) films. The AFM topography image of poly(EDOT-MI-*co*-EDOT-PC) film under different stages. (c) Before the immobilization of the peptide probe. (d) After the thiol-ene click reaction between the maleimide group and peptide probe. (e) After the CaM protein bioconjugated with the probes.

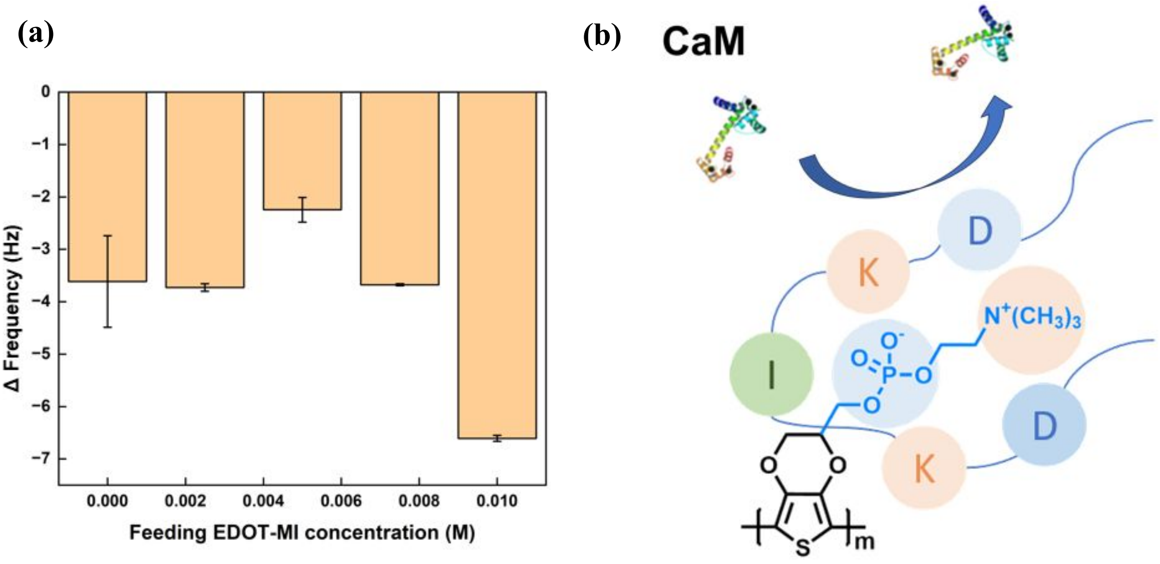

Figure S4. (a) Frequency drop of peptides on different concentrations of poly(EDOT-PC-*co*-EDOT-MI) film. (b) The possible mechanism of peptides adsorbed on phosphorylcholine functional groups.

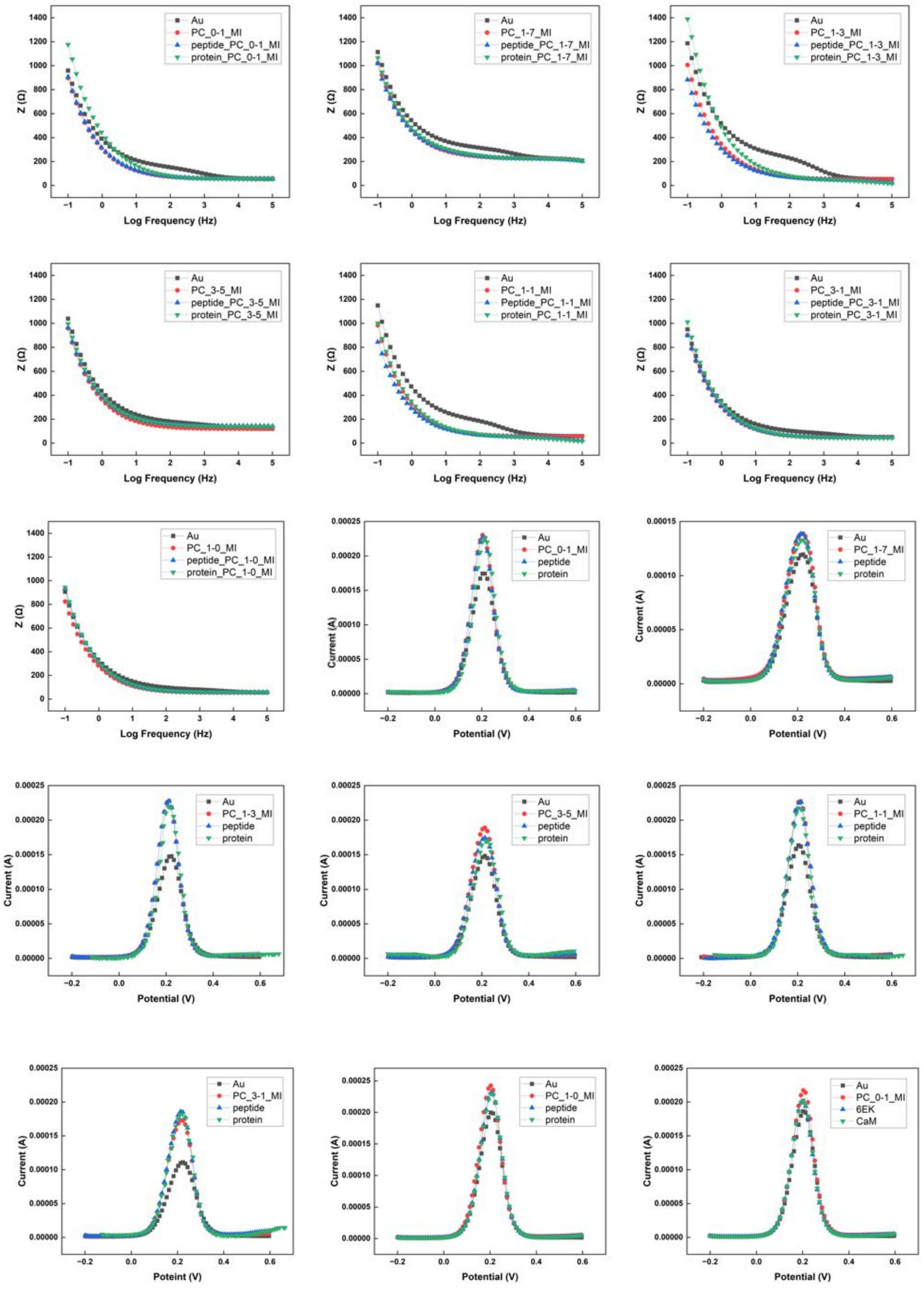

Figure S5. The EIS and DPV measurement on the Au electrode with the copolymer film composed of different feeding ratios of EDOT-PC and EDOT-MI.
